# Insights into the Microbiome of Breast Implants and Periprosthetic Tissue in Breast Implant-Associated Anaplastic Large Cell Lymphoma

**DOI:** 10.1038/s41598-019-46535-8

**Published:** 2019-07-17

**Authors:** Jennifer N. Walker, Blake M. Hanson, Chloe L. Pinkner, Shelby R. Simar, Jerome S. Pinkner, Rajiv Parikh, Mark W. Clemens, Scott J. Hultgren, Terence M. Myckatyn

**Affiliations:** 1 Department of Molecular Microbiology, Washington University School of Medicine, St. Louis, Missouri USA; 2 Center for Women’s Infectious Disease Research, Washington University School of Medicine, St. Louis, Missouri USA; 3 Center for Infectious Diseases, School of Public Health, University of Texas Health Sciences Center, Houston, USA; 4 Center for Antimicrobial Resistance and Microbial Genomics, McGovern Medical School, University of Texas Health Sciences Center, Houston, USA; 5 Department of Epidemiology, Human Genetics & Environmental Sciences, School of Public Health, University of Texas Health Sciences Center, Houston, USA; 6 Division of Plastic & Reconstructive Surgery, Washington University School of Medicine, Alvin J. Siteman Cancer Center, Saint Louis, Missouri USA; 7 Department of Plastic Surgery, The University of Texas MD Anderson Cancer Center, Houston, Texas USA

**Keywords:** Bacterial infection, Surgical oncology

## Abstract

Though rare, breast implant-associated anaplastic large cell lymphoma (BIA-ALCL), a CD30+ T-cell lymphoma associated with textured breast implants, has adversely impacted our perception of the safety of breast implants. Its etiology unknown, one hypothesis suggests an initiating inflammatory stimulus, possibly infectious, triggers BIA-ALCL. We analyzed microbiota of breast, skin, implant and capsule in BIA-ALCL patients (n = 7), and controls via culturing methods, 16S rRNA microbiome sequencing, and immunohistochemistry. Alpha and beta diversity metrics and relative abundance of Gram-negative bacteria were calculated, and phylogenetic trees constructed. *Staphylococcus spp*., the most commonly cultured microbes, were identified in both the BIA-ALCL and contralateral control breast. The diversity of bacterial microbiota did not differ significantly between BIA-ALCL and controls for any material analyzed. Further, there were no significant differences in the relative abundance of Gram-negative bacteria between BIA-ALCL and control specimens. Heat maps suggested substantial diversity in the composition of the bacterial microbiota of the skin, breast, implant and capsule between patients with no clear trend to distinguish BIA-ALCL from controls. While we identified no consistent differences between patients with BIA-ALCL-affected and contralateral control breasts, this study provides insights into the composition of the breast microbiota in this population.

## Introduction

Breast implant-associated anaplastic large cell lymphoma (BIA-ALCL) is a distinct CD30+, anaplastic lymphoma kinase (ALK) negative T-cell lymphoma. The development and progression of this lymphoma is characterized by T cell activation, oligoclonal expansion, and activation of JAK/STAT signaling^1–4^. While BIA-ALCL is relatively rare, impacting an estimated 722 (as of May 14, 2019) women with breast implants worldwide^5^, additional confirmed cases are being reported weekly, and it is has become a major concern among surgeons due to its association with textured, but not smooth, implant surfaces^2,6^. Several purported pathophysiologic mechanisms for developing BIA-ALCL, possibly working in concert, have been put forth, including implant immunogenicity due to silicone or its degradation products from textured implants, a genetic susceptibility rendering a host autoimmune response, an IL-13 associated allergic response, or inflammation triggered by bacterial biofilm^1,2,4,7,8^.

A preliminary report indicated there was an association between bacterial biofilm formation and BIA-ALCL^7,9,10^. Bacterial biofilms may stimulate a robust T-cell response, which may influence BIA-ALCL development^9^. Importantly, textured breast implants harbor more bacteria than smooth-surfaced devices, which may explain why only women who had textured implants developed BIA-ALCL^10–12^. Moreover, *Ralstonia spp*, a Gram-negative microorganism, has been identified in an unexpectedly high proportion of BIA-ALCL specimens^7^. Confirmation of a causative link between bacterial infection and BIA-ALCL, however, remains elusive^7,13^. The breast is a clean-contaminated surgical site, and several studies have successfully identified bacteria on clinically normal breast implants^7,14–16^, suggesting that the mere identification of bacteria on the implant surface does not inherently confirm the presence of pathology. While some bacteria are known to directly cause cancer^17,18^, more often microorganisms are opportunistic and are associated with particular malignancies^19^. Thus, elucidating whether bacterial colonization plays a role in BIA-ALCL remains of critical interest.

In the current study we perform a detailed analysis of the bacterial microbiota across several sites within the implanted breasts of patients with unilateral BIA-ALCL. The goals of this study were to i) determine whether the bacterial species colonizing the BIA-ALCL complicated and contralateral control devices were different and ii) whether those same species could also be found colonizing the breast environment, which may provide insights into how these devices initially become colonized. Differences in microbial diversity, the bacteria present, and the relative abundance of bacteria, including *Ralstonia spp*, were analyzed and compared between BIA-ALCL and contralateral control sides.

## Results

### Clinical correlation

Specimens were collected from July 2017 to May 2018. Patient’s presented with BIA-ALCL at 51.7 ± 11.4 years of age, 9.3 ± 2.0 years after implant placement (Table 1). Breast implants were placed for cosmetic augmentation in 3 patients, and for reconstruction in 4. All patients presented with an effusion, while disease was noted to extend into the capsule in 4 cases. Stage ranged from IA-IIA, and all patients were exclusively treated with *en bloc* resection with complete disease remission to date in all cases (Supplementary Fig. 1).

**Table 1:** Clinical details of BIA-ALCL patients^*^.

Pt#	Age	Reason for Implant	Time to Disease (yrs)	Presentation	TNM Stage	Disease Location	Rx	Outcome
1	36	Cosmetic	8.1	Effusion	IA	Effusion	Complete surgical resection	Complete remission
2	54	Cosmetic	12.2	Effusion	IA	Effusion, capsule	Complete surgical resection	Complete remission
3	47	Recon	6.8	Effusion	IA	Effusion	Complete surgical resection	Complete remission
4	42	Cosmetic	9.2	Effusion	IIA	Effusion, capsule	Complete surgical resection	Complete remission
5	66	Recon	10.3	Effusion	IC	Effusion, capsule	Complete surgical resection	Complete remission
6	51	Recon	7.3	Effusion	IA	Effusion	Complete surgical resection	Complete remission
7	66	Recon	11.0	Effusion	IC	Effusion, capsule	Complete surgical resection	Complete remission

^*^Patient #8 had a double capsule, seroma, and Biocell textured device but not BIA-ALCL. Patient #9 provided breast parenchyma and skin at the time or primary augmentation mastopexy.

### Microbes cultured from patient samples

Coagulase negative staphylococci, including *S*. *epidermidis*, were the most common bacteria isolated and were present in 2 of 7 samples from BIA-ALCL-affected breasts as well as one contralateral control (Table 2). Additionally, *Paenibacillus spp*. was cultured from one ALCL-affected breast and *Lactococcus lactis* was cultured from one non-ALCL breast.

**Table 2:** Bacteria recovered from patient specimens.

Patient #	(+/−) ALCL	Tissue/Material Cultured	Growth in Culture (+/−)	Culture Sequencing	Growth in CFUs (+/−)	Single Colony Sequencing
1	+	Capsule	−		−	
	−	Capsule	−		−	
2	+	Implant, Capsule	− −		− −	
	−	Implant, Capsule	− −		− −	
3	+	Implant, Capsule	− −		− 1×10^0 (aerobic)^	− *Paenibacillus sp*.
4	+	Capsule	−		−	
	−	Implant, Capsule	− −		− −	
5	+	Implant, Capsule	+ +	*Staphylococcus sp*. *(capitis/epidermidis/caprae)* No growth when restruck	− −	
	−	Implant, Capsule	+ −	*Lactococcus lactis*	1×10^0 (anaerobic)^ −	*Staphylococcus epidermidis*
6	+	Implant, Capsule	− −		− −	
	−	Implant, Capsule	− −		− −	
7	+	Implant, Capsule, ADM, Skin, Seroma	− − + − −	*Staphylococcus sp*. *(capitis/epidermidis/caprae)*	− − − − −	
8	Delayed Seroma	Implant, Capsule, Skin, Seroma	− − − −		− − − −	

### Microbial ecology of BIA-ALCL

**Figure 1:**
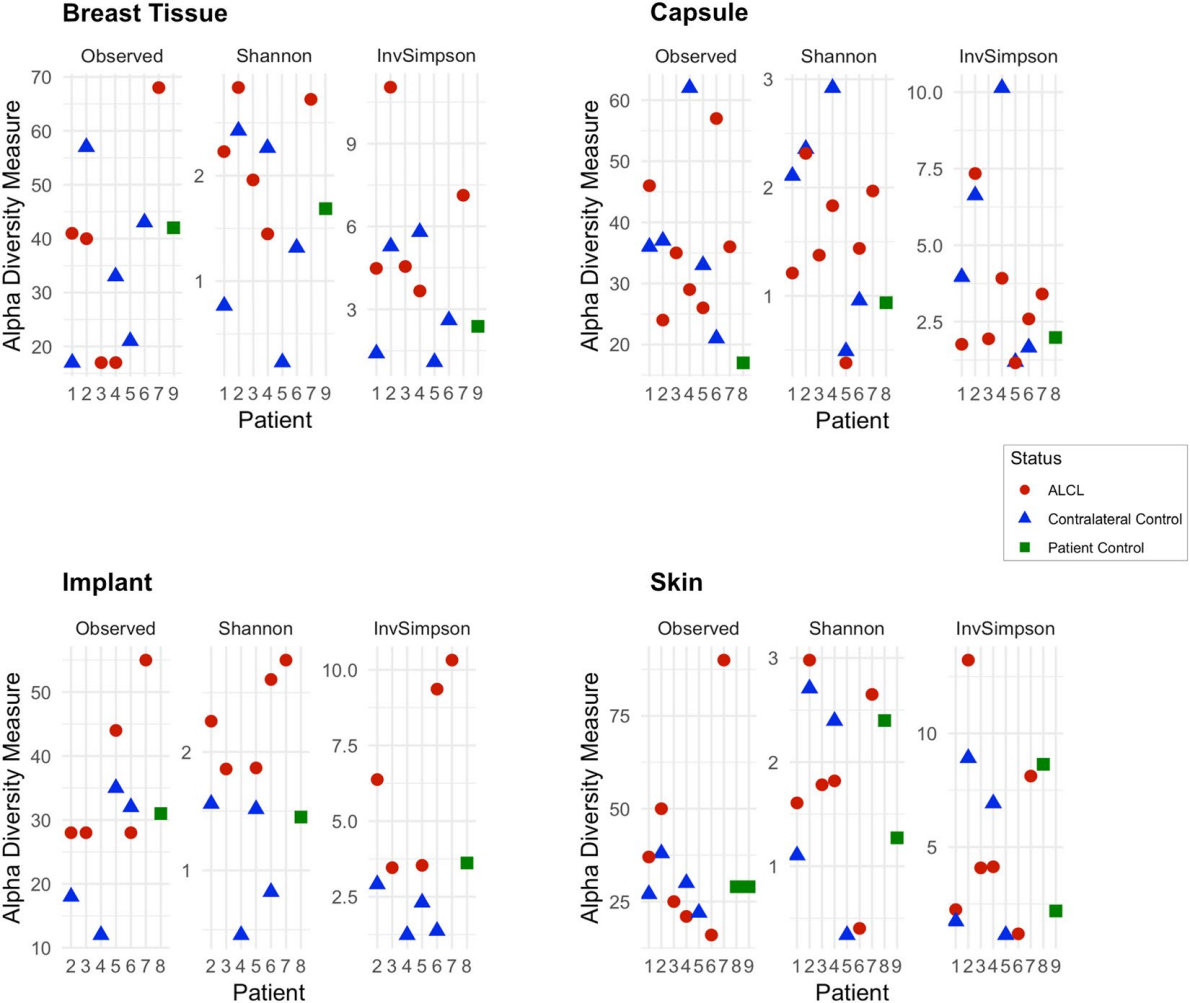
Alpha diversity metrics stratified by BIA-ALCL status and sample type. No statistical difference was identified when comparing BIA-ALCL to the contralateral controls. The Shannon and Inverse Simpson diversity metrics were higher in implant samples from the BIA-ALCL side of patients than the contralateral control.

**Figure 2:**
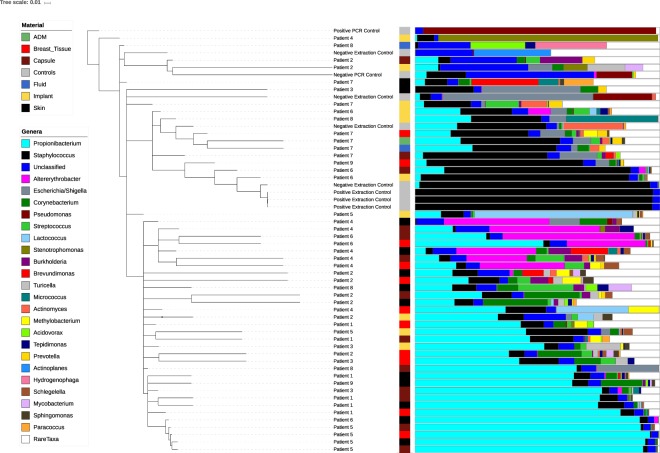
Dendrogram showing sample similarity. Sample clustering was performed using the Bray-Curtis dissimilarity matrix. The top 25 genera across all samples are shown. The stacked bar plot corresponding to each sample shows the relative abundance of the taxa that comprise that sample. Positive and negative extraction controls, as well as positive and negative PCR controls are shown with their respective identified taxa.

**Figure 3:**
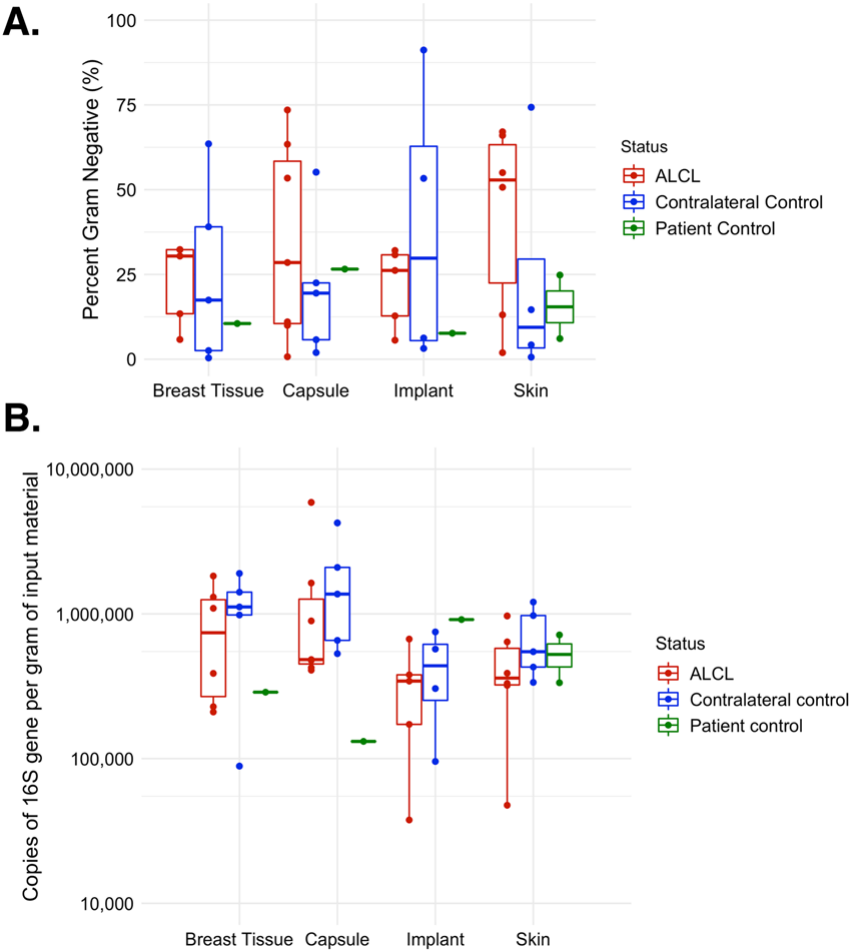
(**A**) Proportion of Gram-negative taxa by sample type and BIA-ALCL status. No difference was found in proportion between BIA-ALCL (red), the contralateral controls (blue), or the non-BIA-ALCL specimens (green) by sample type. (**B**) Quantification of bacteria by sample type and BIA-ALCL status. No difference was found in the quantity of 16S copies between BIA-ALCL (red), the contralateral controls (blue), or the non-BIA-ALCL specimens (green) by sample type.

**Figure 4:**
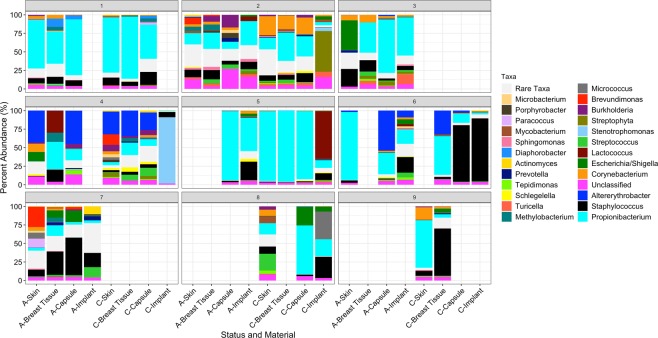
Stacked bar plot of taxa by patient and sample site. The top 25 genera in each sample and the relative abundance of the taxa that comprise the sample are shown for all patient sites. Comparison of BIA-ALCL (**A**) and control (**C**) samples indicate that specimens from within the same patient are generally more similar than samples from other patients, regardless of site or ALCL status.

**Figure 5:**
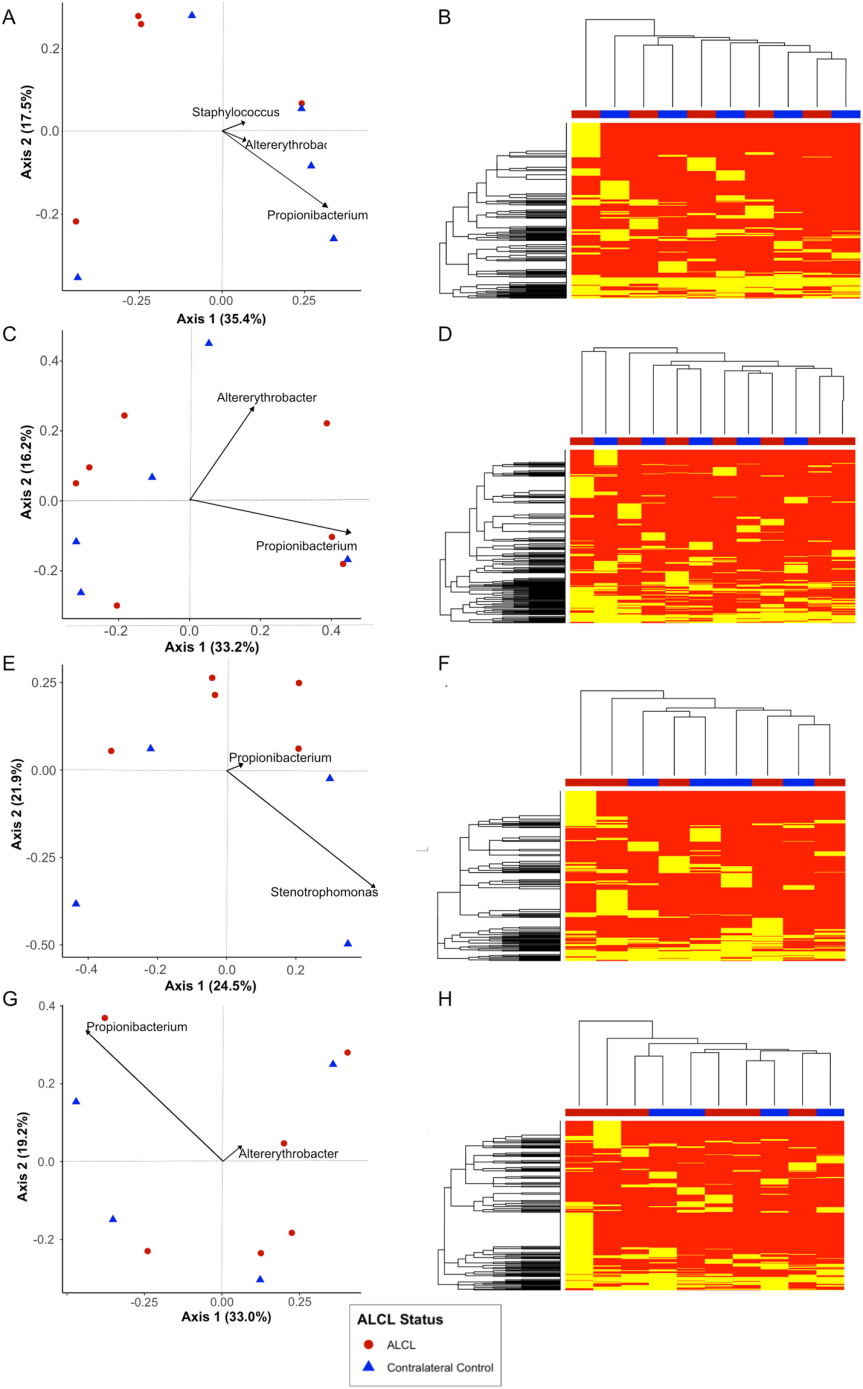
Ordination and heat maps. Ordinations were created using the Bray-Curtis dissimilarity matrix and the top two sources of variability were plotted. Heatmap shows the presence and absence of a taxa as compared across all breast tissue samples. (**A**) Breast tissue ordinations. Overall variability of 52.9% was accounted for in axes 1 and 2. The primary drivers of variability in these samples were *Propionibacterium*, *Staphylococcus*, *and Altererythrobacter*. (**B**) Breast tissue samples (x-axis) are clustered by Euclidian distance, indicating there is not any specific clustering by taxonomic presence/absence. (**C**) Capsule ordinations. Overall variability of 49.4% was accounted for in axes 1 and 2. The primary drivers of variability in these samples were *Propionibacterium and Altererythrobacter*. (**D**) Capsule tissue samples (x-axis) are clustered by Euclidian distance, indicating there is not any specific clustering by taxonomic presence/absence. (**E**) Implant ordinations. Overall variability of 46.4% was accounted for in axes 1 and 2. The primary drivers of variability in these samples were *Stenotrophomonas*, *and Propionibacterium*. (**F**) Implant material samples (x-axis) are clustered by Euclidian distance, indicating there is not any specific clustering by taxonomic presence/absence. (**G**) Skin ordinations. Overall variability of 52.2% was accounted for in axes 1 and 2. The primary drivers of variability in these samples were *Propionibacterium and Altererythrobacter*. (**H**) Skin samples (x-axis) are clustered by Euclidian distance, indicating there is not any specific clustering by taxonomic presence/absence.

Analysis of 16S rRNA microbiome sequencing indicated 49 of the 51 patient samples passed QC, generating more than 1,000 amplicons after filtering (range: 4,995-1,328,993). A wide diversity of bacteria were identified across all samples, accounting for 567 unique genera. The observed, Shannon and Inverse Simpsons’ alpha diversity metrics were calculated and there were no significant differences between samples from the BIA-ALCL breasts and the contralateral controls (Fig. 1). The most abundant genera identified were Propionibacterium, *Staphylococcus*, *Altererythrobacter*, *Escherichia/Shigella*, and *Corynebacterium* (Fig. 2). It is important to note, however, that many of these taxa are observed at high abundance in only a few samples (e.g. *Altererythrobacter* is primarily identified in Patients 4 and 5). While the relative proportion of Gram-negative bacteria ranged from 0.38–91% (a skin and an implant sample, respectively), no significant differences were observed when comparing BIA-ALCL breast samples to contralateral controls (Fig. 3A). Additionally, samples from patients often clustered with other samples from the same patient and show common profiles of the bacteria that make up the microbiota around the breast implant and periprosthetic tissues (Figs 2 and 4). Interestingly, when samples were stratified by material and BIA-ALCL samples were compared to control samples, we found that *Propionibacterium* was the largest driver of variability as calculated using the Bray-Curtis dissimilarity matrix^20^, with *Staphylococcus*, *Altererythrobacter*, and *Stenotrophomonas* also contributing to the observed variability, although not across all sample types (Fig. 5). We also found that while there were a number of rare taxa present within each sample, these genera did not differentiate BIA-ALCL samples from their contralateral controls. Specifically, relative abundance of *Ralstonia* was identified at low abundance within 8 samples (range: 3.3 × 10^−4^–1.6%) and was primarily identified in the non-ALCL controls (2 - ALCL, 5 - Contralateral control, 1 - Cosmetic control). Brevundimonas was also identified in 31 samples at low abundance (range: 7.35 × 10^−4^–27%), with the highest relative abundances at 14.3% and 27.3% for samples from a contralateral control skin and an BIA-ALCL side skin sample, respectively. Importantly, we assessed whether the positive and negative controls clustered with the samples they were extracted with, as well as generated pairwise alignments of the amplified 16S reads to assess for contamination in our samples. The negative extraction controls did not cluster with the batches of samples they were extracted with, and pairwise comparisons of amplified 16S genes showed the bacteria present within the negative controls did not match those identified within our samples (data not shown). Additionally, to quantify the number of bacteria present on BIA-ALCL samples and contralateral controls, 16S qPCR was utilized. There were no significant differences observed between the BIA-ALCL and contralateral control samples or within each tissue type (Fig. 3B).

### Immunofluorescence analysis

**Figure 6:**
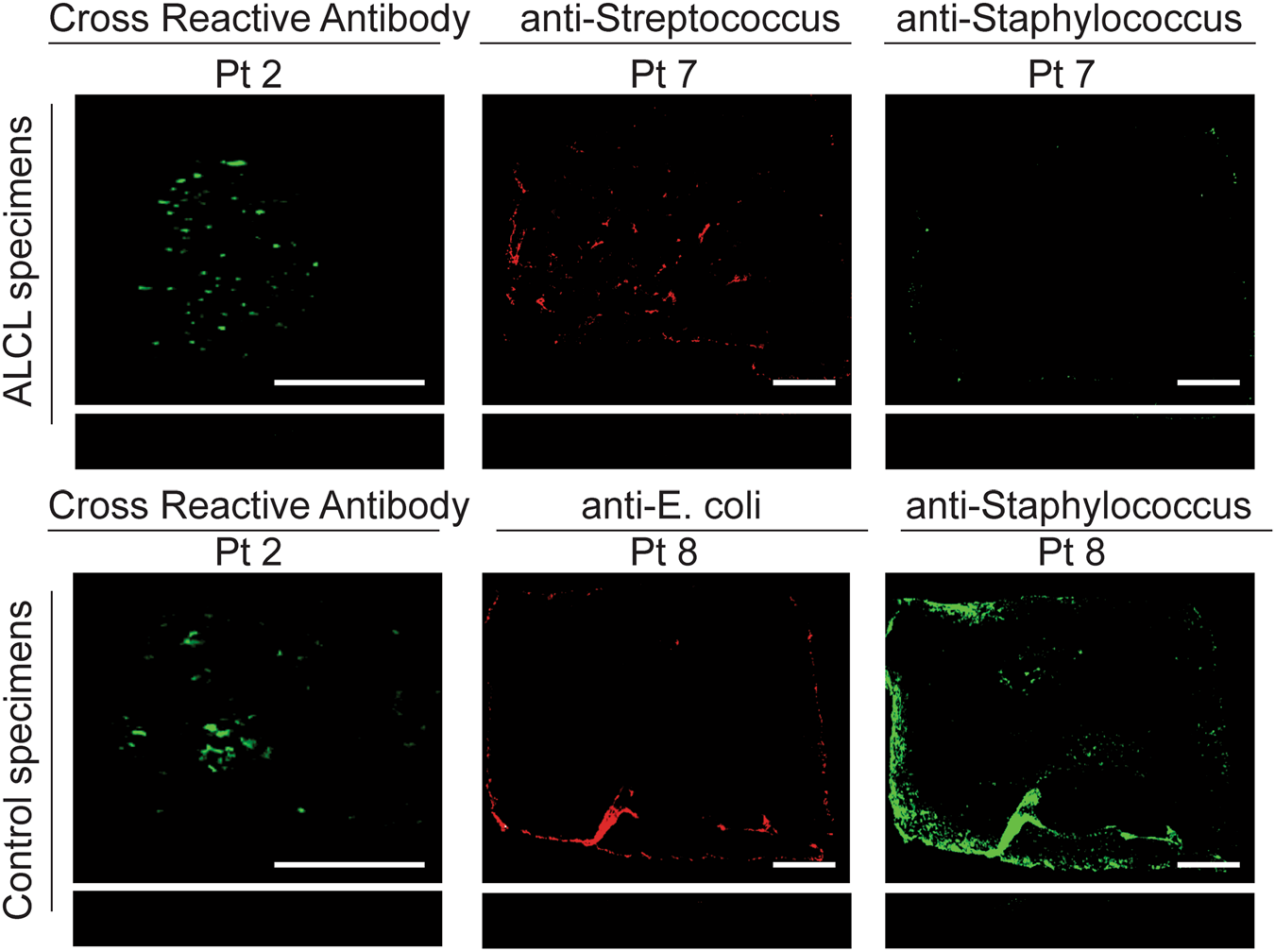
Bacterial identification with immunofluorescence. Using antibodies that cross react with multiple bacteria, bacteria could be detected on a majority of patient samples (7/10) including BIA-ALCL (n = 4) and control (n = 3) specimens. Patient 2’s samples are shown as a representative example. Additionally, patient implants 7 (BIA-ALCL) and 8 (double capsule, delayed seroma, no BIA-ALCL) were stained specifically for *Staphylococci/Streptococci* and *Staphylococci/E*. *coli*, respectively. The bacteria could be detected in both samples. Scale bars denote 0.5 cm.

Immunofluorescence staining determined whether bacteria identified via culturing and/or 16S microbiome sequencing could be visualized on devices (Fig. 6). Due to the large number of microbes identified via 16S microbiome sequencing and the general small size of the specimen, non-specific primary antibodies were used to stain the implant samples to optimize the number of bacteria detected. Bacteria could be detected on the majority (7/10) of breast prostheses, a representative sample of which is shown in Fig. 6. Additionally, due to the large specimen sizes of patient 7 (ALCL) and patient 8 (control with double capsule and delayed seroma but no BIA-ALCL), these samples were also stained for *S*. *epidermidis*/*Streptococcus* and *S*. *epidermidis*/*E*. *coli*, respectively, based on our culturing and 16S microbiome sequencing results. *Staphylococcus* and *streptococcus* could be detected on patient 7’s implant and *Staphylococci* and *E*. *coli* could be detected on patient 8’s implant (Fig. 6).

## Discussion

In this study the bacteria that could be cultured from BIA-ALCL patient specimens were exclusively Gram-positive. *Staphylococcus spp*., including *S*. *epidermidis*, were the predominant bacteria cultured and were present in two specimens from patients with BIA-ALCL and one control patient sample. Furthermore, 16S microbiome sequencing identified *Staphylococcus spp*. in the majority of patient skin, breast, implant and capsular samples analyzed, including the samples from which staphylococci were cultured, indicating this species is likely present and viable on patient implants and within the breast environment. In addition to *Staphylococcus spp*., *Propionibacterium spp*. were also one of the most common bacterial species identified via 16S microbiome sequencing in analyzed patient samples. The identification of *Staphylococci* and *Propionibacterium*, known members of the normal skin microflora, was expected in patient skin specimens^21,22^. Their presence on capsule, implant, and breast tissue specimens supports previous work demonstrating that the bacteria that cause complications may come from the patient’s own microbiome^22,23^. Additionally, the majority of the skin, breast tissue, and capsule specimens display common bacterial taxa within the same patient, regardless of BIA-ALCL status, indicating women more closely mirror themselves than other BIA-ALCL patients (Fig. 4). For example, within Patient 1, we can see the specimens were weighted towards the genus *Propionibacterium* and *Staphylococcus*, with bacterial microbiota composition of the skin, breast, and capsular tissue varying slightly between each sample (Fig. 4). This is consistent with other microbiome studies that generally show patients are more similar to themselves than to other people with the same condition or disease manifestation^24^.

While a previous study implicated certain bacterial species in the development of BIA-ALCL, our analyses fail to identify clear associations between the bacterial microbiome and BIA-ALCL in any tissue-type analyzed^7^. In contrast to this previous work^7^, we noted a relatively low abundance of *R*. *pickettii*. While both our studies had several similarities in methodology, including i) sequencing the V1-V3 region of the 16S gene and ii) using the same taxonomic classifier; our studies differed in that we used Illumina paired-end sequencing in this work while Hu *et al*. used FLX pyrosequencing^7,25^. The use of Illumina sequencing allowed us to assure we covered the full V1-V3 region for all amplicons, as well as implement stringent data quality checks and filters^26^. We also included negative extraction controls and a negative polymerase chain reaction (PCR) control, which is essential to differentiate the kit-ome from the presence of true bacterial species in low biomass samples. It is well established that the negative extraction controls are never truly “negative” within microbiome sequencing, as there is always amplification of bacterial DNA present within the kits used for extraction or during processing^27^. Thus, by assuring the taxa from the “kitome” do not align with the sequencing data from the samples, we were able to differentiate true sequencing data from this low-level amplification from the reagents. Additionally, the technique used for procuring and storing specimens *ex vivo* in advance of analysis can impact 16S rRNA bacterial profile and the generation of OTUs^28–30^, thus specimens collected for 16S microbiome sequencing in our study were immediately stored in 98% EtOH to preserve nucleic acids. Furthermore, to gain a more comprehensive view of the breast enviroment in patients with BIA-ALCL, we collected samples from multiple sites, including the breast tissue, capsule, implant, and skin from both the affected and contralateral control breasts. Hu *et al*. collected a higher number of BIA-ALCL specimens (n = 19), however, all specimens analyzed were capsules and only three contralateral controls were used for comparison analyses^7^. These samples were then used to demonstrate there were higher bacterial burdens in BIA-ALCL compared to the contralateral controls. When we adjusted our quantification data to grams of input material, we did not find any differences in bacterial abundance. Importantly, our results indicate patients are more similar to themselves than to other women with BIA-ALCL, thus it is difficult to know how these differences effect the previously reported results.

In addition to the single species *R*. *picketti*, Gram-negative bacterial-induced inflammation has also become a component of the unifying hypothesis for the development of BIA-ALCL. Bacterial infection and lymphomagenesis has been identified in follicular lymphoma^31^, and mucosal-associated lymphoid tissue^32,33^. Activation of inflammatory pathways such as toll-like receptor (TLR) signaling, of which Gram-negative lipopolysaccharide (LPS) is a TLR-4 ligand, can lead to inflammation-driven cancer progression^34–36^. Additionally, a recent study investigating allergic asthma shows that TLR-4/LPS signaling modulates a leukotriene B4 receptor-2-mediated cascade in mast cells resulting in the release of the Th2 cytokine IL-13^37^. Importantly, IL-13 is produced by anaplastic tumor cells in BIA-ALCL specimens, which also contain mast cells and eosinophils, further linking Gram-negative bacteria and BIA-ALCL^1^. However, the relative abundance of Gram-negative bacteria in our study did not significantly differ between BIA-ALCL skin, breast, capsule, or implant specimens relative to corresponding controls (Fig. 4). Interestingly, Kadin *et al*. note that chronically inflamed, benign breast implant capsules were also positive for IL-13, raising the question of whether this chronic allergic reaction is specific to genetically susceptible patients who ultimately develop BIA-ALCL or equally generalizable to asymptomatic breast implant patients^1^. Together with our data, these studies highlight the need to further evaluate the source of chronic inflammation to determine the role of tumor induction through prolonged antigenic stimulation. Further investigation of the unique features of textured breast implants beyond bacterial contamination, the impact of other infectious agents, or a chronic self-antigen-induced signaling pathway represent additional areas of study that may advance our knowledge of BIA-ALCL^38^.

While our study provides a unique view for the comparison between BIA-ALCL affected breasts and their contralateral controls, there are several limitations. Due to the rarity of this disease we were only able to analyze a small sample size which precluded rigorous statistical analysis. This limitation is amplified by the degree of microbial diversity between specimens and the potential impact of sampling bias both between and within patients^39^. 16S microbiome sequencing provides a measure of relative but not absolute bacterial abundance. Further, it does not provide information on either the viability or metabolic activity of the bacterial species it identifies^29,30^, nor does it differentiate an opportunistic from a causative role^16^. Thus, using 16S microbiome sequencing to determine associations with disease causation has been notoriously difficult^40^. While our data suggests there are no differences between BIA-ALCL affected and contralateral control breasts it is difficult to know whether the composition of the breast microbiome has been stable over the entire time or whether there were significant changes during the transformation to BIA-ALCL years prior that are no longer present in the specimens we characterized in our analysis. While we elected to compare BIA-ALCL specimens to the contralateral side to eliminate the variation bacterial communities can display from one patient to the next, we recognize that the contralateral breast in patients with BIA-ALCL may themselves demonstrate microbial dysbiosis that predisposes to the eventual development of this disease process. Our data, however, demonstrate that bacterial community composition is more strongly associated with a particular patient rather than material sampled or BIA-ALCL diagnosis (Fig. 4)^4,31–34^. Thus, differences in the microbial composition from one patient to the next would confound comparisons of patients with BIA-ALCL to control patients without. A methodologically rigorous study with greater statistical power that includes controls with and without a history of BIA-ALCL would be required to control for variations in bacterial diversity between groups. Going forward, our research, and others, are seeking to provide clarity on this topic through the increased collection and microbiome analysis of samples from patients with and without a history of BIA-ALCL.

## Conclusions

While our study was unable to identify potential correlations between microbial colonization and the development of BIA-ALCL, by assessing specimens from women with unilateral BIA-ALCL and their respective contralateral control, we were able to gain a better understanding of the microbial composition of the skin, breast tissue, capsule, and implant from multiple patients. This data will be invaluable going forward for guiding future studies in this research area, which may provide insights into the development of effective prevention or treatment strategies.

## Methods

### Study population and specimen procurement

Specimens from patients with BIA-ALCL undergoing bilateral explantation and capsulectomy (ie. BIA-ALCL and contralateral control sides) were obtained with institutional review board (IRB) approval from the University of Texas MD Anderson Cancer Center (#PA14-0321). Specimens from patients with double capsules and seromas with and without BIA-ALCL as well as a separate negative control were explanted at the Alvin J Siteman Cancer Center, Saint Louis (IRB #201703063). All patients at both sites underwent informed consented prior to sample collection. All studies were performed in accordance with the regulations and guidelines for the IRB at the University of Texas MD Anderson Cancer Center and the Alvin J Siteman Cancer Center. Detailed clinical information recorded included reason for breast implant, surface texturing, duration of implantation, pathologic stage of BIA-ALCL, and clinical presentation (Table 1).

*En bloc* capsulectomy was performed in all cases of BIA-ALCL. Two to 25 sq cm of aseptically harvested specimens (implant, capsule, skin, and parenchyma) were cut in half with one piece immediately stored in 98% ethanol (EtOH) to preserve nucleic acid integrity for 16S rRNA microbiome sequencing and the other placed in phosphate buffered solution (PBS) for culturing and imaging. Researchers were blinded to the BIA-ALCL etiology and samples were analyzed as described below.

### Sample culturing

Specimens in PBS were divided into three pieces, the largest piece (>0.5 sq. cm) was used for immunofluorescence staining and the two smaller pieces (~2 grams) were cultured. The first piece was submerged in 1 ml of the rich media Brain Heart Infusion (BHI) broth and cultured for ≥24 hours, shaking at 37 °C. For cultures with visible microbial growth, a 10 µL inoculating loop was used to streak for single colonies on BHI agar. Colonies were assessed based on morphology, size, and color and one colony of each type was selected for identification via 16S Sanger sequencing^41^. The remainder was sonicated in 1 ml of PBS for 10 mins, vortexed, and plated on two sets of BHI agar plates, one of which was grown aerobically and the other anaerobically ≥24 hours. If the colonies were homogeneous, colony forming units (CFUs) were recorded and one colony was selected for identification via 16S Sanger sequencing. For heterogeneous colonies, CFUs were recorded for each type based on morphology, size, and color and then a single colony of each was selected for identification via 16S Sanger sequencing.

### Sample processing and DNA extraction

Specimens in EtOH were frozen at −80 °C until DNA extraction. The DNeasy PowerBiofilm kit (Qiagen; Cat # 24000-50) was used for all DNA extractions according to the manufacturers’ instructions; the MP Biomedicals FastPrep-24 Classic Instrument (MP Biomedicals; Cat # MP116004500) was used for the bead beating step. At least one negative extraction control was performed alongside sample extractions to account for any potential kit contaminants.

### 16S rRNA microbiome sequencing and data processing

Amplicons of the V1-V3 regions of the 16S ribosomal RNA (rRNA) gene were amplified for each specimen using custom oligonucleotides containing: unique dual barcodes for each sample, Illumina specific sequencing adapters, and 16S primers for the 27F and 534R bacterial rRNA^42^. Positive (pure *Pseudomonas aeruginosa* gDNA) and negative (pure molecular grade H2O) PCR controls were included to verify amplification capability and ensure the absence of nucleic acid contamination. Purified 16S PCR products were quantified and pooled at equimolar concentration, and sequenced using an Illumina Miseq with the 2 × 300 v3 sequencing kit (Illumina; Cat # MS-102-3003).

Sequencing data was processed as follows: Trimmomatic was used to remove the 16S primers^43^, paired sequences were assembled using PEAR^43^, bbplit (https://sourceforge.net/projects/bbmap/) was used to remove PhiX, and bmtagger was used to remove human DNA sequencing data^44,45^. Remaining amplicons were classified with the RDP Classifier to identify taxonomy^46^. Samples with more than 1,000 amplicons following processing were considered successful.

### 16S rRNA qPCR quantification

Quantitative PCR (qPCR) using an adapted version of the BactQuant protocol was used to quantify the relative number of bacteria within each tissue type^47^. Briefly, the same 16S primers, amplification conditions, and analysis methods were utilized; with the exception that SybrGreen was used instead of a Taqman probe. All samples were run in triplicate, with negative (molecular grade water) and positive controls, including a known standard curve of Escherichia coli 16S genes ranging from 10^2^ to 10^7^. The number of 16S copies identified was adjusted for the elution volume of each sample, and standardized by the amount of input material in grams, generating the copies of the 16S gene per gram of input material.

### Data analysis

All investigators except MWC were blinded to BIA-ALCL versus control side in all cases where bilateral specimens were provided until raw data were collated for analysis. All analyses of the 16S rRNA microbiome data were completed with R version 3.4.2. The phyloseq package was used to assess alpha diversity for each sample using the observed diversity, Shannon Index, and the Inverse Simpson Index^48^. Data was rarified to 4,955 amplicons per sample (the lowest number of amplicons in a sample that passed QC) prior to calculating alpha diversity to normalize the number of reads for each sample. Reads were not normalized for any other analyses to maintain the highest precision possible for each sample^49^. Additionally, beta diversity was assessed using the Bray-Curtis dissimilarity and depicted with ordination plots using principal coordinates analysis using the ggplot2 and vegan packages^50,51^. Box and whisker plots comparing the relative abundance of Gram-negative bacteria were plotted using ggplot2. Presence/absence of operation taxonomic units (OTUs) in BIA-ALCL and non-BIA-ALCL samples was represented using binary heatmaps for each sample type and plotted using gplots^52^. Phylogenetic trees were plotted using the Interactive Tree of Life software v3^53^. Statistical differences in the relative abundance of Gram-negative bacteria and 16S copy numbers per gram of input material between BIA-ALCL samples and the contralateral controls were assessed using the Wilcoxon rank sum test. Analyses compared samples stratified by material, and were completed in two manners: (i) accounting for all samples, both matched pairs of BIA-ALCL and contralateral controls from a single patient and samples where only the BIA-ALCL sample was available, and (ii) only those samples where the matched pairs of BIA-ALCL and contralateral controls were present.

### Immunofluorescence staining and imaging

The largest implant pieces in PBS were fixed in 10% neutral buffered formalin for 1–2 hours and immunofluorescently stained as previously described^41^. Briefly, implants were blocked overnight at 4 °C in 1.5% bovine serum albumin and 0.1% sodium azide in PBS and then washed with PBS-T (PBS containing 0.05% Tween-20). Primary antibodies were diluted 1:500 in dilution buffer (0.05% Tween, 0.1% bovine serum albumin, and 0.5% methyl alpha-D-mannopyranoside in PBS) and incubated with the implants for 2 hrs. Implants were then washed with PBS-T and incubated with secondary antibodies diluted 1:10,000 in dilution buffer for 45 min. Implants were washed again with PBS-T and examined for infrared signal using the Odyssey Imaging System (LI-COR Biosciences; Cat# B446). Negative controls included implant pieces treated identically to the above, with the exception that the primary antibody was not included.

### Antibodies used

Primary antibodies used were mouse: anti-Gram positive bacteria antibody (*Staphylococcus*, *Enterococcus*, *Bacillus*, *Listeria*, and *Streptococcus spp*.*)* (Abcam; Cat # ab20344), anti-eEF1A1/EF-Tu antibody (*Bacteroides*, *Streptococcus*, *Bacillus*, *Pseudomonas*, *Burkholderia*, and *Deinococcus*) (Abcam; Cat # ab90813), and anti-*Staphylococcus epidermidis* (coagulase negative staphylococci) (ThermoFisher Scientific; Cat # MA1-35788)*;* rabbit: anti-*Escherichia coli* antibody (*E*. *coli*) (US Biological; Cat# E3500-04K) and anti-Streptococcus group D antigen (*Streptococcus spp.*) (Lee Laboratories). Secondary antibodies used were: IRDye 800CW donkey anti-mouse (LI-COR Biosciences; Cat# 926-32212) and IRDye 680LT donkey anti-rabbit (LI-COR Biosciences; Cat# 926-68023).

## Supplementary information

Supplementary Figure 1

## Data Availability

Datasets generated and analyzed in the current study are available under NCBI BioProject ID PRJNA512850. Clinical datasets used in this study are not publicly available as the data is highly sensitive but are available from the corresponding author on reasonable request.
