# Supplementary material for: Insights into the Microbiome of Breast Implants and Periprosthetic Tissue in Breast Implant-Associated Anaplastic Large Cell Lymphoma
Source: Sci Rep. 2019 Jul 17;9:10393. doi: 10.1038/s41598-019-46535-8 (PMC6637124; doi:10.1038/s41598-019-46535-8)
Supplement: Supplementary file 1 — Supplementary Figure 1 [file 41598_2019_46535_MOESM1_ESM.pdf]

Insights into the Microbiome of Breast Implants and Periprosthetic Tissue in Breast Implant-Associated Anaplastic Large Cell Lymphoma

Jennifer N. Walker, PhD; Blake M. Hanson, PhD; Chloe L. Pinkner, BA; Shelby R. Simar, BA; Jerome S. Pinkner, MS; Rajiv Parikh, MD, MPHS; Mark W. Clemens, MD, FACS; Scott J. Hultgren, PhD; Terence M. Myckatyn MD, FACS, FRCSC

Supplementary Figure 1 – Clinical Case of BIA-ALCL. A) Patient #7 presented with a delayed seroma 11.0 years after insertion of breast implant to restore symmetry in a healthy breast. Contralateral breast had undergone prosthetic post-mastectomy breast reconstruction for Stage IIB breast adenocarcinoma. Aspirated seroma fluid confirmed diagnosis with CD30+, ALK- T-lymphocytes. Intraoperative fluid aspiration (shown here) verified diagnosis. B) Explanted form stable gel implant with BioCell textured surface. C) Explanted capsule following *en bloc* resection. D) CD30+ T-lymphocytes (40x magnification).

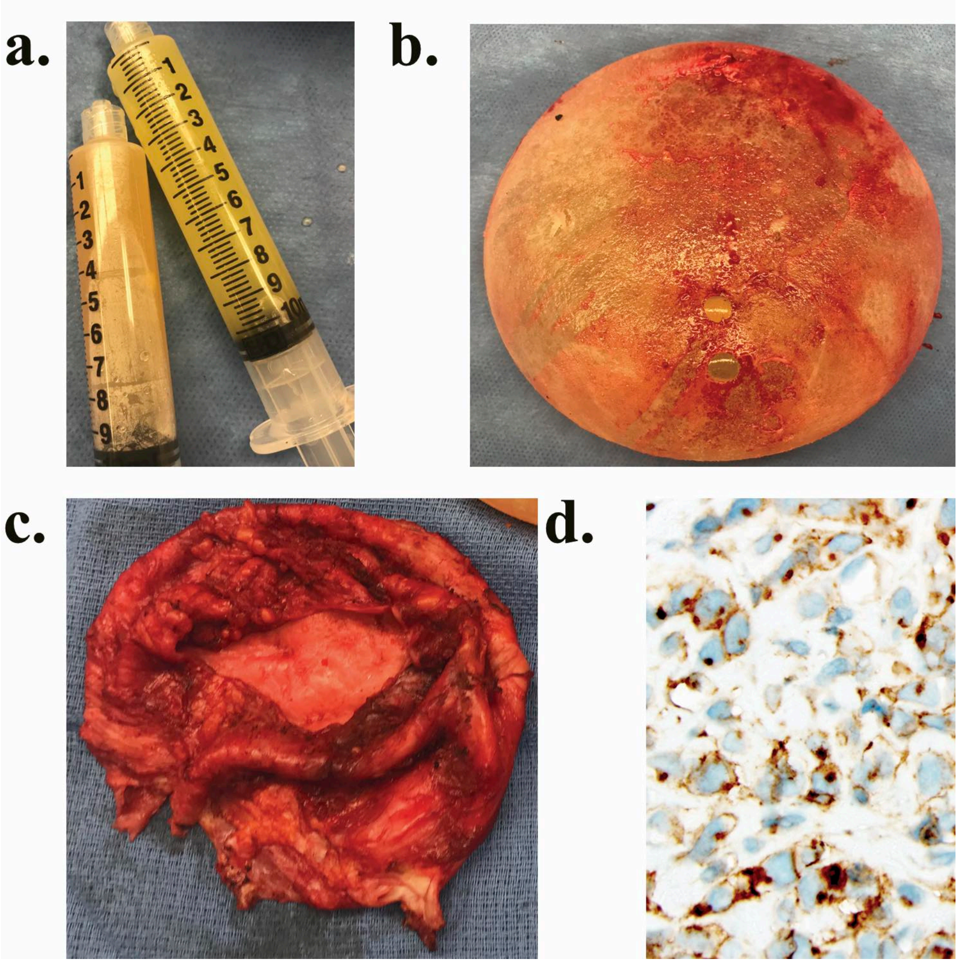
a.
b.
c.
d.
